# Generalized gingival enlargement, periodontitis, and osteonecrosis of the jaw as manifestations of IgG4-related disease: A rare case report

**DOI:** 10.1097/MD.0000000000043260

**Published:** 2025-07-11

**Authors:** Jie Zhao, Yufeng Ling, Lingying Niu, Lei Zhang, Liangyuan Lin, Juan Wu

**Affiliations:** a Nanjing Stomatological Hospital, Affiliated Hospital of Medical School, Research Institute of Stomatology, Nanjing University, Nanjing, China; b Department of Rheumatology and Immunology, Nanjing Drum Tower Hospital, The Affiliated Hospital of Nanjing University Medical School, Nanjing, China; c Department of Stomatology, Children’s Hospital of Nanjing Medical University, Nanjing, China.

**Keywords:** case report, gingival enlargement, IgG4-related disease, osteonecrosis of the jaw, periodontitis

## Abstract

**Rationale:**

Immunoglobulin G4-related disease (IgG4-RD) is an immune-mediated systemic fibro-inflammatory condition involving one or multiple organs. We report a rare case of IgG4-RD associated with generalized gingival enlargement, periodontitis, and osteonecrosis of the jaw in a Chinese man.

**Patient concerns:**

A 44-year-old Chinese man visited the Department of Periodontology with gingival enlargement and increasing tooth mobility. He had previously undergone nonsurgical periodontal treatment and surgical periodontal treatment. However, his symptoms were not relieved.

**Diagnoses:**

The patient had extremely poor oral hygiene and a large amount of calculus. The gingivae were edematous with deep periodontal pockets and attachment loss, and the mandible contained localized alveolar bone necrosis. Panoramic radiography revealed severe alveolar bone loss. Serologic examination revealed that the level of IgG4 concentration was 199 mg/dL. Histopathological findings demonstrated significant lymphoplasmacytic infiltration. The immunohistochemical staining showed 50 scattered IgG4-positive plasma cells in a high-power field, and the IgG4/IgG cell ratio was >50%. The patient was finally diagnosed with IgG4-RD.

**Interventions:**

He then received multidisciplinary treatments: glucocorticoid treatment, nonsurgical periodontal treatment, sequestrectomy, and curettage. His gingival enlargement significantly improved without recurrence.

**Outcomes:**

His gingival enlargement significantly improved without recurrence.

**Lessons:**

Patients with generalized gingival enlargement and periodontitis who do not seem to respond to conventional periodontal therapy must be suspected of having periodontitis associated with systemic disease, such as IgG4-RD.

## 1. Introduction

Immunoglobulin G4-related disease (IgG4-RD) is a chronic, systemic, immune-mediated fibroinflammatory disease involving one or multiple organs, including the pancreas, liver, kidneys, salivary glands, lymph nodes, orbits, meninges, retroperitoneum, mediastinum, pericardium, and aorta.^[1]^ Manifestations of IgG4-RD have been demonstrated in nearly every organ system. The involved organs share similar clinical, pathological, and serological features, including fibrous sclerosing tumors or hypertrophic lesions, lymphoplasmacytic infiltrate enriched in IgG4-positive plasma cells, and a variable degree of fibrosis with a characteristic storiform pattern.^[2]^ Additionally, 60% to 70% of patients with IgG4-RD have elevated serum concentrations of IgG4.^[3]^ The most common manifestations of IgG4-RD include type 1 autoimmune pancreatitis, IgG4-related sclerosing cholangitis, major salivary gland enlargement and sclerosing sialadenitis, inflammatory orbital pseudotumor, chronic sclerosing dacryoadenitis, retroperitoneal fibrosis, chronic sclerosing aortitis and periaortitis, IgG4-related interstitial pneumonitis, and IgG4-related kidney disease.^[4]^

Notably, lesions affecting the oral cavity have been infrequently reported, and oral manifestations of IgG4-RD are often neglected in most reviews on this theme. Consequently, clinicians often fail to recognize IgG4-related oral lesions, which lead to misdiagnosis and unnecessary surgical interventions.^[5]^ In a large single-center cohort of Italian patients, IgG4-RD localizations in the oral cavity were observed in 6 of 200 patients with oral lesions (3%), including mass-forming lesions at the hard palate, superior alveolar processes, tonsillar and peritonsillar region, tongue base extending to the oropharynx, and tongue base.^[6]^ A scoping review of IgG4-RD cases involving the oral cavity revealed that there were 51 cases of oral IgG4-RD in the literature. The hard palate and jaw bones were the 2 main locations reported.^[7]^ Here, we report a rare case of IgG4-RD with generalized gingival enlargement, periodontitis, and osteonecrosis of the jaw (ONJ) that met the 2020 revised comprehensive diagnostic (RCD) criteria for IgG4-RD.^[8]^

## 2. Case presentation

**Figure 1.**
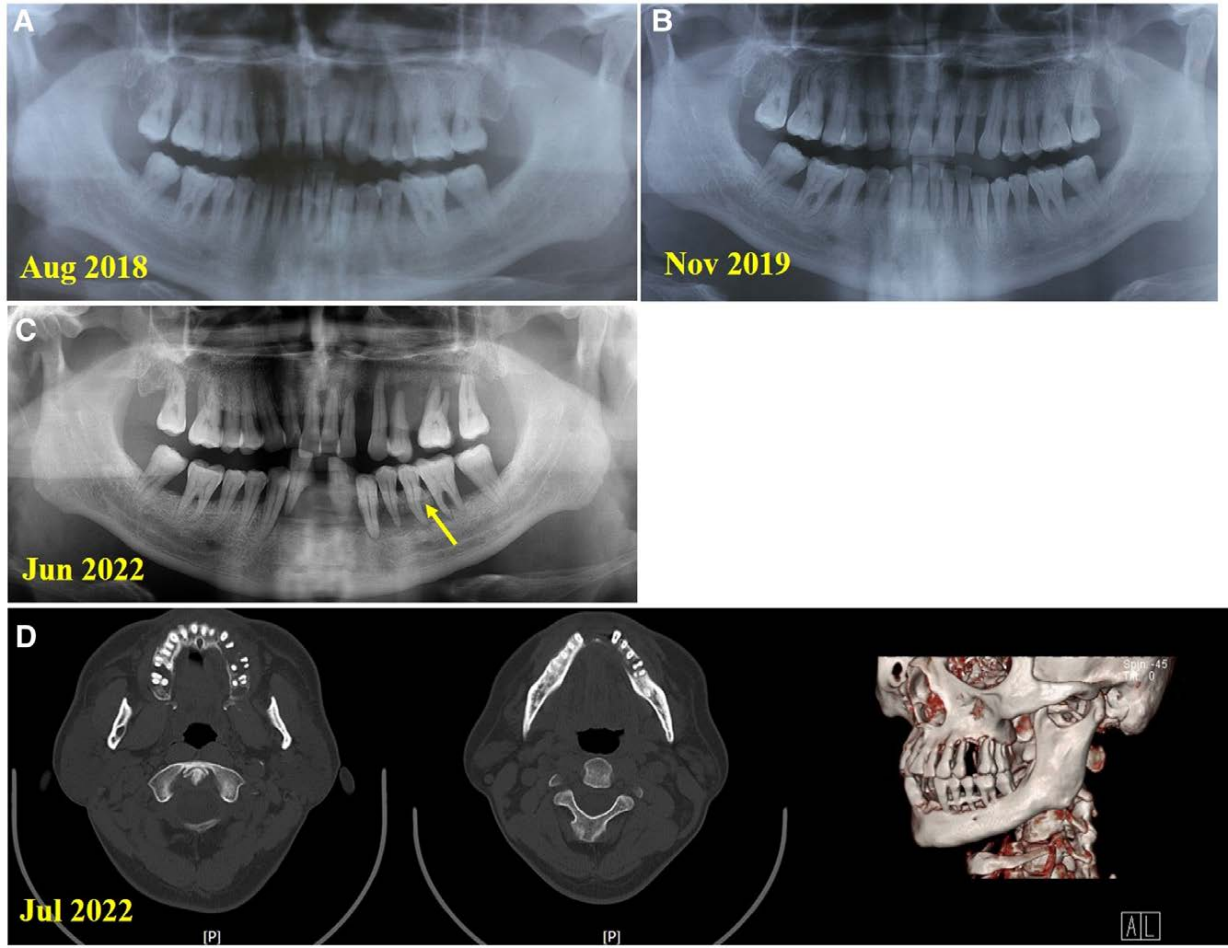
Orthopantomogram and spiral computed tomography images of the patient. (A, B) Images obtained at different times demonstrate rapidly progressing alveolar bone resorption and missing teeth. (C, D) Alveolar bone necrosis on teeth 33 to 35. Yellow arrow: Alveolar bone necrosis.

**Figure 2.**
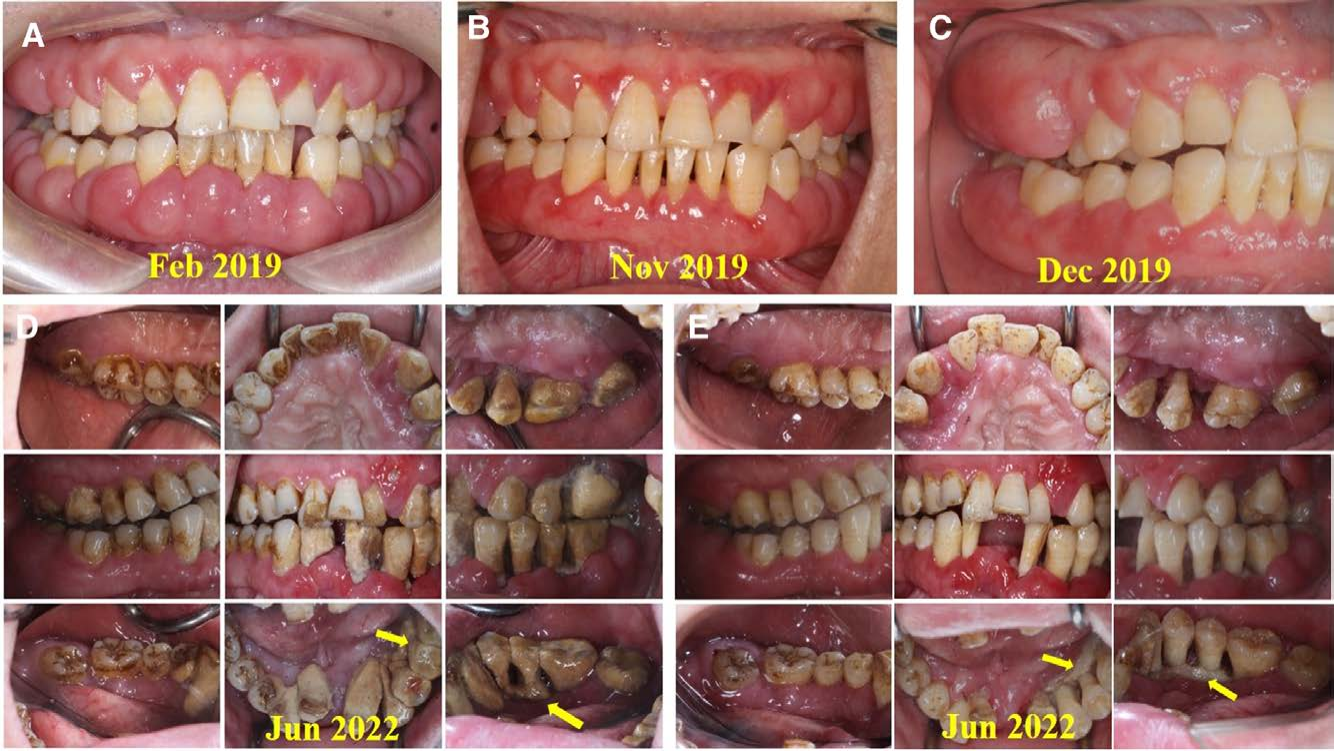
Oral photographs of the patient taken at different times. (A, B, C) Oral photographs taken before he was referred to our hospital. (D, E) Oral photographs taken after he was referred to our department. (A) Generalized gingival enlargement (February 2019) that appeared spherical, sessile, and involved marginal and attached gingiva and papilla. The involved gingivae were edematous, rubbery, pink, soft, elastic, and bled easily. (B, C) At 6 and 7 mo, after periodontal flap surgery and gingivectomy on teeth 34 to 47, significant gingival hyperplasia recurrence was noted on teeth 43 to 47. (D) Intraoral photographs of the patient at first examination: oral hygiene was extremely poor, with a mass of dental plaque and calculus covering almost all remaining teeth. Generalized gingival enlargement presented as dusky red, swollen, soft, and bled easily. (E) After most of the supragingival and subgingival calculus had been removed, intraoral examination revealed localized alveolar bone necrosis in the mandible (teeth 33–35 area). Yellow arrow: Alveolar bone necrosis.

A 44-year-old Chinese man was first referred to the Department of Periodontology, Nanjing Stomatological Hospital in June 2022. At the initial examination, he complained of generalized gingival enlargement and increased tooth mobility for 5 years. In 2017, he felt that his gums were obviously swollen and hyperplastic after moving to a new house. He consulted the local hospital in August 2018. The orthopantomogram (OPG) revealed apparent alveolar bone resorption (Fig. 1A). He underwent hematological investigation, including complete blood count, coagulation, and liver and renal function, and the test results were within normal limits. The immune function tests (IgG, IgA, IgM, C3, C4, immunoglobulin E (IgE), erythrocyte sedimentation rate [ESR]) revealed elevated serum IgE (>1000 IU/mL, reference range: 0–100 IU/mL) and ESR (71 mm/h, reference range: <15 mm/h). However, no allergens were identified. He was diagnosed with periodontitis, gingival hyperplasia, and plasma cell gingivitis (PCG). He underwent nonsurgical periodontal treatment (NSPT), including supragingival scaling, subgingival scaling and root planing (SRP). However, the gingival hyperplasia was not relieved, and he was referred to another hospital in February 2019. At the second hospital, it was determined that he had poor oral hygiene with generalized gingival enlargement, especially in the facial and buccal region (Fig. 2A). The gingival enlargement appeared spherical, sessile, and involved marginal and attached gingiva and papilla. The involved gingiva was edematous, rubbery, pink, soft, elastic, and bled easily. The gingival margin was inflamed, with no ulceration or significant necrosis. Periodontal examination revealed generalized bleeding on probing, deep periodontal pockets, and attachment loss. He was also diagnosed with periodontitis and PCG. Subsequently, the patient underwent SRP, periodontal flap surgery, and gingivectomy under local anesthesia (teeth 34–47 in March 2019, teeth 14–17 in December 2019). The biopsy revealed massive collagen fiber deposition and chronic inflammation with lymphocytes and plasma cell infiltrate. Following the periodontal flap surgery and gingivectomy on teeth 34 to 47, the patient attended a 6- and 7- month follow-up. However, significant gingival hyperplasia recurrence was noted on teeth 43 to 47 (Fig. 2B, C). The OPG revealed increased alveolar bone resorption (Fig. 1B). Subsequently, he returned to the local hospital and underwent gingivectomy (teeth 21–27 and 31–37) under general anesthesia in February 2021. However, the gingival hyperplasia recurred rapidly, and he was referred to our hospital in June 2022. There was no relevant family history, and he has seasonal eczema. His previous medical history revealed no significant causative factors of gingival enlargement.

Clinical examination: Extraoral examination suggested facial symmetry without any obvious abnormalities in the lymph nodes of the head and neck. His oral hygiene was extremely poor, with a mass of dental plaque and calculus covering almost all the remaining teeth (Fig. 2D). The severe periodontitis was accompanied by generalized gingival enlargement surrounding all teeth, especially in the mandibular anterior and left mandibular posterior tooth areas. The involved gingivae were dusky red, swollen, soft, and bled easily. The gingival margin was inflamed with no ulceration or significant necrosis. Periodontal examination revealed generalized bleeding on probing, extremely deep periodontal pockets, and severe attachment loss. Teeth 25, 31, and 41 fell out. Tooth mobility was I^°^–III^°^ and was as follows: tooth 43, grade I^°^; teeth 17, 22, 23, 33, and 37, grade II^°^; and teeth 24, 26, 27, 32, 34, 35, 36, and 42, grade III^°^. There were no oral mucosal lesions. The major salivary glands, including the parotid, submandibular, and sublingual glands, were not enlarged. After removing most of the supragingival and subgingival calculus, we detected localized alveolar bone necrosis (sequestrum) in the mandible (teeth 33–35 area). The sequestrum was linked to teeth 33 to 35 and partially separated from the underlying healthy alveolar bone (Fig. 2E).

Radiographic findings: The OPG revealed generalized and severe alveolar bone loss, especially in the left posterior tooth area, with rapidly progressing alveolar bone resorption extending to the root apex (Fig. 1C). Spiral computed tomography 3-dimensional reconstruction revealed alveolar bone loss with a fuzzy or worm-eaten appearance on the teeth 33 to 36 and 43 to 46 areas, and tooth 33 to 35 had alveolar bone necrosis (Fig. 1D).

Serological examination revealed high serum IgG4 (199 mg/dL), IgE (780 IU/mL), and ESR (29 mm/h). The other blood tests (complete blood count, coagulation, liver and renal function, C-reactive protein, C3, C4, IgA, IgG, IgM, and autoimmune disease testing) were all within normal limits. Antinuclear antibodies and antineutrophil cytoplasmic antibodies (ANCA) were negative, while anti-myeloperoxidase antibody, anti-proteinase 3 antibody and anti-glomerular basement membrane antibody were all within normal ranges (Table 1).

**Table 1:** Hematochemical findings in the present patient described in this paper.

Blood parameter	The patient	Reference range	UoM
Hemochromocytometric test			
RBC count	4.44	4.3–5.8	10^12^/L
Hemoglobin	**125**	130–150	g/L
Hematocrit	**38.7**	40–50	%
MCV	87.2	82–100	fl
MCH	28.2	27–34	pg
MCHC	327	316–354	g/L
RDW-CV	**15.8**	0–14	%
Platelets	163	125–350	10^9^/L
WBC count	6.3	3.5–9.5	10^9^/L
Lymphocytes	1.3	1.1–3.2	10^9^/L
Monocytes	0.6	0.1–0.6	10^9^/L
Neutrophils	4.0	1.8–6.3	10^9^/L
Eosinophils	0.35	0.02–0.52	10^9^/L
Basophils	0.04	0–0.06	10^9^/L
Inflammatory indices			
ESR	**29**	<21	mm/h
CRP	4.7	0–8	mg/L
C3	1.31	0.8–1.6	g/L
C4	0.30	0.2–0.4	g/L
Serum protein electrophoresis			
Albumin	44.9	40–50	g/L
Albumin/globulin ratio	1.47	1.2–2.4	–
Immunoglobulins			
IgE	**780**	0–100	IU/mL
IgG	1050	800–1600	mg/dL
IgG4 subclass	**199**	8–140	mg/dL
Autoantibodies			
ANA antibodies	Neg	Neg	Neg/Pos
ENA	Neg	Neg	Neg/Pos
p-ANCA	Neg	Neg	Neg/Pos
c-ANCA	Neg	Neg	Neg/Pos
Anti-MPO	<20	0–20	Ru/mL
Anti-PR3	<20	0–20	Ru/mL
Anti-GBM	<20	0–20	Ru/mL
Anti-SM antibody	Neg	Neg	Neg/Pos
Anti-MPO	<20	0–20	Ru/mL
Anti-PR3	<20	0–20	Ru/mL
Anti-GBM	<20	0–20	Ru/mL
Anti-SM antibody	Neg	Neg	Neg/Pos
Anti-Sc1-70 antibody	Neg	Neg	Neg/Pos
Anti-PM-Sc1 antibody	Neg	Neg	Neg/Pos
Anti-Jo-1 antibody	Neg	Neg	Neg/Pos
Anti-CENP-B antibody	Neg	Neg	Neg/Pos
Anti-PCNA antibody	Neg	Neg	Neg/Pos
Anti-dsDNA antibody	Neg	Neg	Neg/Pos
AnuA	Neg	Neg	Neg/Pos
AHA	Neg	Neg	Neg/Pos
Anti-AMA-M2 antibody	Neg	Neg	Neg/Pos
Anti-SSA antibody	Neg	Neg	Neg/Pos
Anti-SSB antibody	Neg	Neg	Neg/Pos
Anti-Ro-52 antibody	Neg	Neg	Neg/Pos

Bolded values are those outside the normal ranges.

AHA = anti-histone antibody, AMA = anti mitochondrial antibody, ANA = antinuclear antibody, ANCA = anti-neutrophil cytoplasmic antibodies, Anti-CENP-B = anti-centromere protein B antibody, Anti-GBM = anti-glomerular basement membrane antibody, Anti-MPO = anti-myeloperoxidase antibody, Anti-PR3 = anti-proteinase 3 antibody, Anti-Ro-52 = anti-52 kDa Ro protein-specific antibody, Anti-SM = anti-smith antibody, CRP = c-reactive protein, ENA = extractable nuclear antigens, ESR = erythrocyte sedimentation rate, IgE = immunoglobulin E, IgG = immunoglobulin G, MCH = mean corpuscular hemoglobin, MCV = mean cell volume, PCNA = proliferating cell nuclear antigen, SSA = Sjögren’s Syndrome-antigen A, SSB = Sjögren’s Syndrome-antigen B, RBC = red blood cell, RDW-CV = red blood cell distribution width-coefficient of variation, WBC = white blood cell.

**Figure 3.**
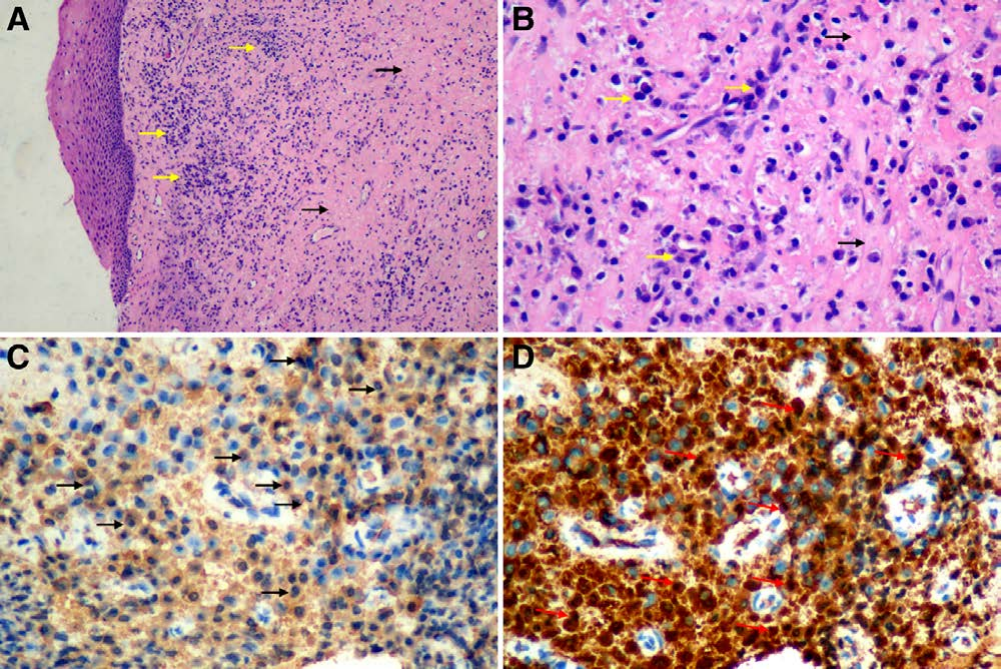
Pathological features of gingival tissue. (A, B) H&E staining: Lymphoplasmacytic infiltration (yellow arrows) with storiform fibrosis (black arrows). (A: 100×; B: 400×); (C) IgG4 staining: numerous IgG4-positive plasma cells are present (black arrows), the plasma cells predominantly expressed IgG4 (IgG4; 50/HPF, 400×); (D) IgG staining: IgG-positive plasma cells (red arrows), the ratio of IgG4/IgG plasma cell was >46.2% (400×). H&E = hematoxylin and eosin staining, IgG = immunoglobulin G.

Histological features: Gingival biopsy was performed on the gingiva during tooth extraction (32 and 42). The hematoxylin and eosin staining results demonstrated nonspecific chronic inflammatory conditions: hyperplastic stratified squamous epithelium and dense lymphoplasmacytic infiltrate enriched with plasma cells accompanied by fibrous hyperplasia typified by a cartwheel appearance of the arranged fibroblasts and inflammatory cells, characterized by storiform fibrosis (Fig. 3A, B). Immunohistochemical staining demonstrated the infiltration of a predominance of IgG4-positive plasma cells in the gingiva, with 50 IgG4-positive plasma cells present per high-power field at 400× magnification (Fig. 3C). The IgG4/IgG cell ratio was >50% (Fig. 3D). Other immunohistochemical markers, including CD3, CD20, Kappa/Lambda, and Ki67 were examined, and neoplasms were excluded.

The diagnosis of IgG4-RD relies on the combination of characteristic histopathologic, clinical, serologic, and radiologic findings. The 2020 RCD criteria specify 3 comprehensive diagnostic criteria for IgG4-RD: clinical and radiological features: ≥1 organs have diffuse or localized swelling or a mass or nodule characteristic of IgG4-RD. Lymph node swelling is omitted in single-organ involvement; serological diagnosis: serum IgG4 > 135 mg/dL; and pathological diagnosis: positivity for 2 of the following 3 criteria: dense lymphocyte and plasma cell infiltration with fibrosis; the ratio of IgG4-positive plasma cells/IgG-positive cells >40% and IgG4-positive plasma cells >10 per high-power field; typical tissue fibrosis, particularly storiform fibrosis, or obliterative phlebitis. Under the standard diagnostic criteria, the finding of all 3 criteria indicates a definite diagnosis, criteria clinical and radiological features and serological diagnosis indicate a probable diagnosis; and clinical and radiological features and pathological diagnosis indicate a possible diagnosis.^[8]^ Subsequently, the patient was referred to the Department of Rheumatology and Immunology, Nanjing Drum Tower Hospital, and a diagnosis of IgG4-RD was made (RCD criteria: “definite”). He then underwent positron emission tomography–computed tomography imaging. Fortunately, there were no changes in other organs.

The differential diagnosis of IgG4-RD typically depends on the specific involvement site and clinical presentation.^[9]^ Differential diagnosis of gingival enlargement requires a thorough dental and medical history; careful evaluation of the type, nature, and extent of enlargement; and the identification of etiologic or predisposing factors.^[10]^ Several diseases associated with gingival enlargement should be considered and ruled out, including inflammatory gingival enlargement, drug-induced gingival enlargement, genetic disorders associated with gingival enlargement, and leukemia. Additionally, IgG4-RD involvement of gingival enlargement should be distinguished from PCG and granulomatosis with polyangiitis (GPA).^[11–13]^ Due to the dense plasma cell infiltration in the gingiva, other physicians initially diagnosed him with PCG. Notably, plasma cell lesions in the oral cavity encompass a wide spectrum ranging from reactive to immune‑mediated and neoplastic. PCG is a rare gingival condition appearing as generalized erythema and edema of the attached gingiva. Diagnostic workup is based on the patient’s history and clinicopathologic correlation, including a full blood count (leukemia), ESR (lupus, infection, inflammation), serum angiotensin converting enzyme (sarcoidosis), ANCA (GPA), and dermatologic patch testing for contact allergens.^[11]^ Unlike IgG4-RD, PCG is characterized pathologically by plasma cell infiltration but generally without storiform fibrosis, and obliterative phlebitis.^[12]^ PCG may show the presence of IgG4+ positive plasma cells, but in these conditions the IgG4/IgG ratio is commonly below the threshold of 40%.^[14]^ In addition, IgG4 related gingival enlargement should be distinguished from GPA, which is a noninfectious small-to medium vessel vasculitis. Up to 5% of affected patients have strawberry gums, which are the initial disease manifestation in 2% of cases.^[15]^ The classic histologic findings of strawberry gums demonstrate pseudoepitheliomatous hyperplasia, epithelial micro-abscesses, multinucleated giant cell infiltrate, and necrotizing vasculitis; furthermore, an eosinophilic and plasma cell–rich inflammation might be detected. Differential Laboratory test results demonstrate an elevated titer of ANCA with a cytoplasmic staining pattern (c-ANCA), and a positive enzyme-linked immunosorbent assay for anti-proteinase 3 antibody.^[16]^

**Figure 4.**
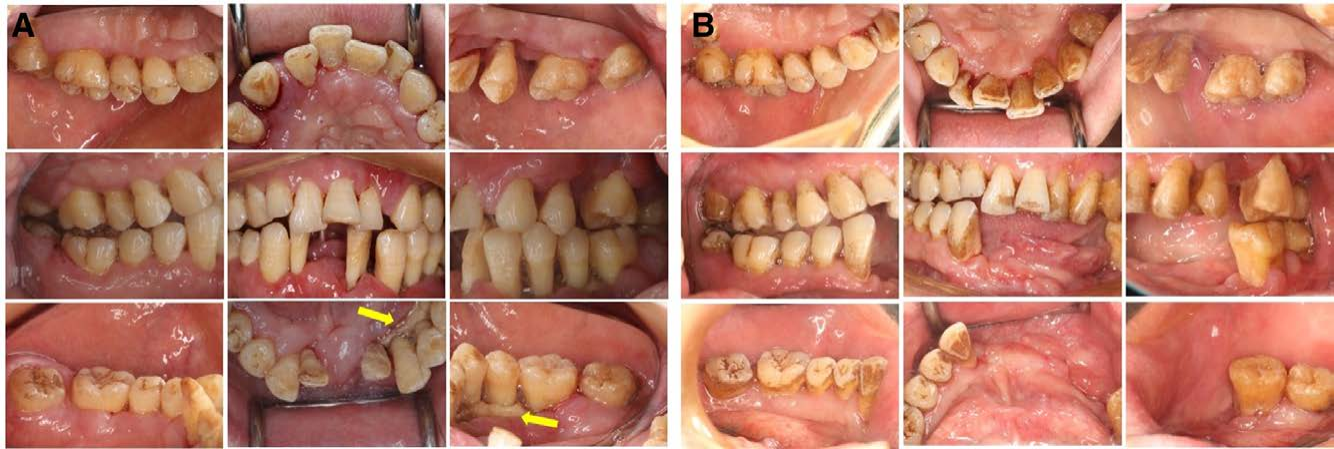
Clinical presentation of gingival changes during treatment. (A) The color of the gingivae turned pink, and gingival enlargement was alleviated 1 wk after SRP in conjunction with 7-d systemic administration of amoxicillin and metronidazole. Yellow arrow: Alveolar bone necrosis and (B) the gingival swelling was significantly improved and the gingival edema disappeared 3 mo after multidisciplinary comprehensive treatment.

Treatment: Before the diagnosis was confirmed, the patient only underwent NSPT and the extraction of tooth 32 and 42. One week after SRP in conjunction with 7-day systemic administration of amoxicillin and metronidazole, the color of the gingivae turned pink, and the gingival enlargement was alleviated (Fig. 4A). Once the diagnosis of IgG4-RD had been established, the patient underwent multidisciplinary treatment programs guided by a rheumatologist. First, the rheumatologist recommended oral glucocorticoid treatment, and the initial prednisolone dose was 0.6 mg/kg for 3 weeks (30 mg/d). The patient also received NSPT, including oral hygiene instruction and SRP. At the 3-week follow-up, the gingivae swelling had significantly improved, and his ESR was normal (10 mm/h). Subsequently, the patient underwent local curettage to remove the necrotized bone in the mandible and extraction of teeth 33 to 35 under general anesthesia. Three weeks after the surgical interventions (Fig. 4B), the gingiva of the remaining teeth turned pink, the gingival enlargement disappeared, and the periodontal pockets became shallow. The patient received continuous glucocorticoid treatment with prednisolone (10 mg/d) for 3 months. At follow-up, his ESR was within the normal range (14 mm/h), and the gingival hyperplasia did not recur. Following this, the rheumatologist recommended a supportive dose of 5 mg/d prednisolone and revisit every 3 months. The periodontist suggested that the patient undergo SRP every 3 months and receive removable dentures to replace his missing teeth. However, the patient failed to adhere to regular follow-ups. No recurrence of gingival hyperplasia was demonstrated when he returned to our department 9 months after the surgical interventions (Fig. S1, Supplemental Digital Content, https://links.lww.com/MD/P391), and his ESR was remained normal (12 mm/h).

## 3. Discussion

Gingival enlargement, also known as gingival overgrowth, refers to an increase in the size of the gingiva and is considered a type of periodontal disease. It is classified based on etiology and pathologic changes into: inflammatory (acute or chronic); drug-induced (e.g., phenytoin, nifedipine, cyclosporine); associated with systemic conditions (e.g., pregnancy, puberty, vitamin C deficiency, leukemia, granulomatous diseases); neoplastic (benign or malignant tumors); and false enlargements.^[17]^ This condition can present as either localized or generalized and is graded in severity as: grade 0: no enlargement); grade I: involvement confined to the interdental papilla; grade II: involvement extending to both the interdental papilla and the marginal gingiva; and grade III: enlargement covering 3-quarters or more of the tooth crown.^[18]^ Here, we report a rare clinical presentation in a patient with IgG4-RD, predominantly manifesting as generalized gingival enlargement with a severity of grade II.

IgG4-RD was first described in 2001 in patients with sclerosing cholangitis with elevated serum IgG4 levels in Japan.^[19]^ The exact prevalence of IgG4-RD is unknown, although a community-based study reported an IgG4-RD incidence of 0.28 to 1.08/1,00,000 population, with 336 to 1300 newly diagnosed patients per year in Japan.^[20]^ Most IgG4‑RD cohorts demonstrate a slight predominance of middle‑aged and older men with variable gender distribution, although IgG4-RD also occurs in children.^[20,21]^ Patients often present with subacute development of a mass in the affected organ or diffuse enlargement of an organ. Head and neck IgG4-RD is the second most common site of presentation after the pancreas, and the most affected areas are the submandibular and parotid glands, orbit, and thyroid.^[22]^ However, IgG4-RD with periodontium involvement is extremely rare. The present report contributes to the limited literature on IgG4-RD with periodontium by detailing the case of a 44-year-old man exhibiting gingival enlargement, periodontitis, and ONJ. The patient exhibited features consistent with IgG4-RD, including generalized gingival swelling or gingival enlargement, elevated serum IgG4 concentration (199 mg/dL, defined as >135 mg/dL), gingival lesions with marked lymphoplasmacytic infiltration, storiform fibrosis, and significantly increased IgG4-positive plasma cells (50 IgG4-positive plasma cells/HPF; IgG4/IgG plasma cell ratio was >50%). Unfortunately, due to the lack of clinical experience and the omission of careful clinical, radiographic, and histopathological examinations, the patient was initially misdiagnosed and underwent unnecessary periodontal surgery between 2018 and 2021 in other hospitals. His condition continued to worsen, resulting in irreversible alveolar bone resorption and tooth loosening. The patient was compelled to continue seeking treatment until he was diagnosed with IgG4-RD at our department.

Additionally, a literature search was performed through the PubMed database using the terms “IgG4-related,” “case,” “oral,” and “gingiva.” Only 9 documented cases with periodontium involvement are available from PubMed so far.^[7,23–29]^ Table 2 summarizes these cases, including ours, where the male/female ratio was 9:1, and the patients’ ages were 30 to 79 years (mean: 53.6 years). Four cases were from China, 4 cases were from Japan, 1 case was from Poland and 1 case was from Italy. All cases except case 9 presented with gingival swelling and alveolar bone destruction, and case 9 presented as PCG. No patient had used any drug that might have caused gingival hyperplasia. Three cases presented generalized gingival enlargement, and 6 cases presented localized gingival enlargements. In 3 cases, the main manifestations were swelling gingiva and inflammatory granulation tissue in the unhealed socket after tooth extraction. Two edentulous patients with localized gingival enlargements were diagnosed with plasma cell granuloma and underwent surgical excision. The tissue specimens obtained during surgery were reevaluated in a retrospective study, and the results indicated 1 definite and 1 probable case of IgG4-RD.^[22]^ Only 3 cases demonstrated IgG4-RD involvement in other organs. All 6 cases who received glucocorticoid therapy demonstrated marked improvement of the symptoms and signs. Surgical periodontal therapy did not improve the gingival hyperplasia in 2 cases with generalized gingival enlargement until they were diagnosed with IgG4-RD. Then, they received glucocorticoid treatment and nonsurgical periodontal therapy, and the gingival hyperplasia improved and did not recur.

**Table 2:** Cases of IgG4-related periodontium.

No	Author	Sex/age/country	Clinical features	Systemic disease	Other related organs	Serological tests		Pathological findings		Treatment
						IgG (mg/dL)	IgG4 (mg/dL)	IgG4+/HPF	IgG4/IgG	
1	Ono K^[23]^	Male/65/Japan	Redness and swelling gingiva in the tooth extraction unhealed socket in the right maxilla, destruction of maxillary bone on the right side	No	The orbit, cheek, lung	N/D	171	135	N/D	Surgical excision before IgG4-RD diagnosis. Orbit and cheek swelling improved after glucocorticoid treatment
2	Laco J^[24]^	Male/79/Japan	Asymptomatic swelling gingiva in lower left (tooth 33–34 area, edentulous jaws)	No	No	N/D	165	139	72%	Surgical excision before IgG4-RD diagnosis. No recurrence at 11-mo follow-up
3	Laco J^[24]^	Male/74/Japan	Asymptomatic swelling gingiva in upper left (tooth 21–23 area, edentulous jaws)	No	No	N/D	N/D	66	71%	Surgical excision before IgG4-RD diagnosis. No recurrence at 20-mo follow-up
4	Gontarz M^[25]^	Male/30/Poland	Swelling gingiva, inflammatory granulation tissue in the tooth sockets (25–26), loosening of tooth 24	No	Cervical lymph nodes and ethmoid sinuses	N/D	335	N/D	80% cervical lymph nodes	Glucocorticoid treatment with good response
5	Tong AC^[26]^	Female/46/China	Unhealed sockets of teeth 44 and 45, with flabby, edematous and verrucous gingiva, loosening of tooth 43	No	No	1410	88	60	>40%	Glucocorticoid treatment, mobility of tooth 43 decreased. No recurrence at 18-mo follow-up
6	Zhang J^[27]^	Male/38/China	Periodontitis, gingival enlargement	No	N/D	2370	280	10	N/D	Glucocorticoid treatment and nonsurgical periodontal therapy. Gingival enlargement was improved without recurrence at 6-mo follow-up
7	Zhu XZ^[28]^	Male/34/China	Periodontitis, gingival enlargement	Iron-deficient anemia, allergic dermatitis	N/D	1650	171	>100	N/D	Gingivectomy before IgG4-RD diagnosis, but gingival hyperplasia did not improve. Subsequently, glucocorticoid treatment and nonsurgical periodontal therapy were received, and gingival hyperplasia improved at 1-mo follow-up
8	Ike Y^[29]^	Male/60/Japan	Ossifying fibrous epulis and bone resorption in the left mandible	Reflux esophagitis	Submandibular lymph nodes	2025	312	45	62.3%	Surgery, No recurrence at 7-yr follow-up
9	Azzi L^[7]^	Male/66/Caucasian	A bright red, mildly granular, erythematous appearance of the upper and lower gums, resembling PCG	No	No	N/D	195	>50	>75%	The gingiva were treated with a combination of topical clobetasol, pimecrolimus, and doxycycline. After 1 mo, the gingival lesions were healed. A mild relapse after 2 mo was managed with topical clobetasol
10	This case	Male/44/China	Generalized gingival enlargement, periodontitis, ONJ	Seasonal eczema	No	1050	199	50	46.2%	Surgical periodontal therapy before IgG4-RD diagnosis, but the gingival hyperplasia did not improve. Glucocorticoid treatment and nonsurgical periodontal therapy were subsequently received, and gingival hyperplasia improved without recurrence at 6-mo follow-up

HPF = high-power field, IgG = immunoglobulin G, N/D = no data, ONJ = osteonecrosis of the jaw, PCG = plasma cell gingivitis.

Currently, the etiology of IgG4-RD remains unclear, although it is thought to be related to genetic background, bacterial infection and environmental factors, and allergies. Environmental exposures, especially of occupational origin, appear to be involved in the development of different types of IgG-RD. A positive history of blue-collar work increases the risk of developing IgG4-RD, especially IgG4-related idiopathic retroperitoneal fibrosis, and mineral dust and asbestos were the most strongly associated industrial compounds.^[30]^ Takanashi et al recently reported an intriguing case of IgG4-RD involving house dust allergy that improved spontaneously without immunosuppressive therapy after the patient moved to another house, suggesting that allergy is closely associated with IgG4-RD pathogenesis and that avoiding allergen exposure can improve IgG4-RD.^[31]^ IgG4-RD patients often have allergy-related diseases, such as asthma, rhinosinusitis, or rhino-conjunctivitis, or skin diseases, such as atopic dermatitis.

Interestingly, we initially determined that the patient’s serum IgE level was significantly elevated (780 IU/mL). As a major mediator of clinical allergy, an elevated serum IgE level was detected in 35.0% to 88.9% of IgG4-RD patients.^[32,33]^ A large retrospective cohort study that investigated serum IgE levels in the clinical features and outcomes of IgG4-RD revealed that IgG4-RD patients with high serum IgE levels at baseline were more likely to have higher disease activity, and baseline high IgE levels were associated with disease relapse.^[34]^ Our patient’s medical history indicated that environmental exposures might have been involved in the development of IgG-RD. The patient felt that his gums were obviously swollen and hyperplastic after moving to a new house, and his serum IgE levels were always high (2018: >1000 IU/mL; 2022: 780 IU/mL) before he was diagnosed with IgG4-RD.

The diagnosis of IgG4-RD is based on the combination of characteristic histopathologic, clinical, and serologic findings. The common clinical features of IgG4-RD are swelling, fibrosis, and sclerosis of the affected organs. Histologic examination of biopsy specimens is the gold standard for diagnosing IgG4-RD, and the pathological features of IgG4-RD are fibroinflammatory masses, storiform fibrosis, and IgG4-positive plasma cell infiltration. A scoping review focusing on published cases of IgG4-RD involving the oral cavity determined that the histological identification of a IgG4/IgG plasma cell ratio ≥40% was central to diagnosis. Conversely, the pathological features of storiform fibrosis and obliterative phlebitis were not common.^[7]^ Our patient met diagnostic criteria, and his symptoms gradually relieved after glucocorticoid therapy, which also supported the IgG4-RD diagnosis.

All patients with symptomatic, active IgG4-RD require treatment. Glucocorticoids are the first-line agent for remission induction in all patients with active, untreated IgG4-RD.^[35]^ Additionally, rituximab is recommended for patients who are resistant to glucocorticoids alone or unable to reduce their dose sufficiently.^[36]^ IgG4-RD treatment involving gingival enlargement should be guided by a rheumatologist. Furthermore, glucocorticoid treatment and NSPT are recommended, but not surgical periodontal therapy. Moreover, careful systemic follow-up is required to screen other organs involved. Our treatments significantly improved the clinical symptoms of the patient, who said, “I’m grateful I persisted in seeking answers. Learning that I had IgG4-RD (a disease I’d never heard of) was a turning point. The treatments have greatly reduced my gum swelling. I hope more clinicians recognize this condition to prevent delayed diagnosis.”

Unlike other cases involving periodontium, our patient also had local ONJ in addition to gingival enlargement and periodontitis. Several systemic and local risk factors correlate with the high frequency of ONJ development.^[37,38]^ The general risk factors include malignancies, chemotherapy, glucocorticoid treatment, and high-dose or long-term bisphosphonate treatment. The local risk factors include maxillary or mandibular bone surgery (tooth extraction and dental implants), poor oral hygiene, chronic inflammation (periodontitis), and ill-fitting dentures. Whether IgG4-RD involves bone tissue remains uncertain. Yim et al reported a 70-year-old man with IgG4-RD presenting as otogenic skull base osteomyelitis.^[39]^ However, Table 2 lists 3 cases in which IgG4-RD presented as unhealed socket and alveolar bone resorption after tooth extraction, suggesting that IgG4-RD might increase the risk of ONJ. We are uncertain whether periodontitis, periodontal surgery, IgG4-RD, or a combination of all 3 caused our patient’s ONJ.

## 4. Conclusion

IgG4-RD frequently presents diagnostic challenges for clinicians. Early recognition and diagnosis are clinically important to prevent irreversible organ damage. Patients with generalized gingival enlargement and periodontitis who do not seem to respond to conventional periodontal therapy must be suspected of having periodontitis associated with systemic disease, such as IgG4-RD. The combination of serological, histological, and clinical features can be a clue for further investigation.

## Author contributions

**Conceptualization:** Liangyuan Lin.

**Funding acquisition:** Juan Wu.

**Investigation:** Jie Zhao, Yufeng Ling, Lingying Niu, Lei Zhang.

**Project administration:** Liangyuan Lin, Juan Wu.

**Software:** Lei Zhang.

**Writing – original draft:** Jie Zhao, Yufeng Ling.

**Writing – review & editing:** Lingying Niu, Liangyuan Lin, Juan Wu.

## Supplementary Material



## References

[1] Kamisawa T Zen Y Pillai S Stone JH. IgG4-related disease. Lancet. 2015;385:1460–71. 25481618 10.1016/S0140-6736(14)60720-0

[2] Umehara H Okazaki K Masaki Y. Comprehensive diagnostic criteria for IgG4-related disease (IgG4-RD), 2011. Mod Rheumatol. 2012;22:21–30. 22218969 10.1007/s10165-011-0571-z

[3] Carruthers MN Khosroshahi A Augustin T Deshpande V Stone JH. The diagnostic utility of serum IgG4 concentrations in IgG4-related disease. Ann Rheum Dis. 2015;74:14–8. 24651618 10.1136/annrheumdis-2013-204907

[4] Wallace ZS Naden RP Chari S. The 2019 American College of Rheumatology/European league against rheumatism classification Criteria for IgG4-Related Disease. Arthritis Rheumatol. 2020;72:7–19. 31793250 10.1002/art.41120

[5] Kouwenberg WL Dieleman FJ Willems SM Rosenberg AJWP. Inflammatory pseudotumour of the alveolar process of the maxilla as clinical manifestation of IgG4-related disease: a case report and literature review. Int J Oral Maxillofac Surg. 2020;49:722–5. 31864897 10.1016/j.ijom.2019.11.008

[6] Rampi A Lanzillotta M Mancuso G Vinciguerra A Dagna L. IgG4-related disease of the oral cavity. case series from a large single-center cohort of italian patients. Int J Environ Res Public Health. 2020;17:8179. 33167472 10.3390/ijerph17218179 PMC7663930

[7] Azzi L Magnoli F Krepysheva D. The “great imitator”: IgG4-related disease of the oral cavity. Two case reports and scoping review. Head Neck. 2024;46:1510–25. 38566594 10.1002/hed.27763

[8] Umehara H Okazaki K Kawa S. The 2020 revised comprehensive diagnostic (RCD) criteria for IgG4-RD. Mod Rheumatol. 2021;31:529–33. 33274670 10.1080/14397595.2020.1859710

[9] Nizar AH Toubi E. IgG4-related disease: case report and literature review. Auto Immun Highlights. 2015;6:7–15. 26216362 10.1007/s13317-015-0069-3 PMC4536235

[10] Agrawal AA. Gingival enlargements: differential diagnosis and review of literature. World J Clin Cases. 2015;3:779–88. 26380825 10.12998/wjcc.v3.i9.779 PMC4568527

[11] Kaur H Mishra D Roychoudhury A Bhalla AS Ramteke PPS Kumar L. Plasma cells in oral lesion: a clue to diagnosis or a diagnostic dilemma. J Oral Maxillofac Pathol. 2022;26:591. 37082057 10.4103/jomfp.jomfp_398_21 PMC10112081

[12] Younis RH Georgaki M Nikitakis NG. Plasma cell gingivitis and its mimics. Oral Maxillofac Surg Clin North Am. 2023;35:261–70. 36805902 10.1016/j.coms.2022.10.003

[13] Leuci S Coppola N Adamo N. Clinico-pathological profile and outcomes of 45 cases of plasma cell gingivitis. J Clin Med. 2021;10:830. 33670562 10.3390/jcm10040830 PMC7922699

[14] Cottom H Mighell AJ High A Bateman AC. Are plasma cell-rich inflammatory conditions of the oral mucosa manifestations of IgG4-related disease? J Clin Pathol. 2015;68:802–7. 26056156 10.1136/jclinpath-2014-202814

[15] Marzano AV Fanoni D Berti E. Oral and cutaneous findings are valuable diagnostic aids in Wegener’s granulomatosis. Eur J Intern Med. 2010;21:49. 20122615 10.1016/j.ejim.2009.09.013

[16] Comarmond C Cacoub P. Granulomatosis with polyangiitis (Wegener): clinical aspects and treatment. Autoimmun Rev. 2014;13:1121–5. 25149391 10.1016/j.autrev.2014.08.017

[17] Hasan S Khan NI Reddy LB. Leukemic gingival enlargement: Report of a rare case with review of literature. Int J Appl Basic Med Res. 2015;5:65–7. 25664273 10.4103/2229-516X.149251 PMC4318106

[18] Goyal A Rathore M Singh SK Nada R. Generalised gingival enlargement as a sole manifestation of IgG4-related disease. BMJ Case Rep. 2020;13:e236338. 10.1136/bcr-2020-236338 PMC7368500 32675130

[19] Hamano H Kawa S Horiuchi A. High serum IgG4 concentrations in patients with sclerosing pancreatitis. N Engl J Med. 2001;344:732–8. 11236777 10.1056/NEJM200103083441005

[20] Umehara H Okazaki K Masaki Y. A novel clinical entity, IgG4-related disease (IgG4RD): general concept and details. Mod Rheumatol. 2012;22:1–14. 21881964 10.1007/s10165-011-0508-6 PMC3278618

[21] Wallace ZS Deshpande V Stone JH. Ophthalmic manifestations of IgG4-related disease: single-center experience and literature review. Semin Arthritis Rheum. 2014;43:806–17. 24513111 10.1016/j.semarthrit.2013.11.008

[22] Takano K Yamamoto M Takahashi H Himi T. Recent advances in knowledge regarding the head and neck manifestations of IgG4-related disease. Auris Nasus Larynx. 2017;44:7–17. 27956101 10.1016/j.anl.2016.10.011

[23] Ono K Shiiba M Yoshizaki M. Immunoglobulin G4-related sclerosing inflammatory pseudotumors presenting in the oral cavity. J Oral Maxillofac Surg. 2012;70:1593–8. 21978720 10.1016/j.joms.2011.07.017

[24] Laco J Kamarádová K Mottl R. Plasma cell granuloma of the oral cavity: a mucosal manifestation of immunoglobulin G4-related disease or a mimic? Virchows Arch. 2015;466:255–63. 25522952 10.1007/s00428-014-1711-6

[25] Gontarz M Wyszyńska-Pawelec G Zapała J Gałązka K Tomaszewska R Lazar A. IgG4-related disease in the head and neck region: report of two cases and review of the literature. Pol J Pathol. 2016;67:370–5. 28547965 10.5114/pjp.2016.65871

[26] Tong AC Ng IO Lo MC. Immunoglobulin G4-related sclerosing disease involving the mandible. Hong Kong Med J. 2017;23:534–6. 29026050 10.12809/hkmj154733

[27] Zhang J Zhao L Zhou J Dong W Wu Y. Immunoglobulin G4-related periodontitis: case report and review of the literature. BMC Oral Health. 2021;21:279. 34049546 10.1186/s12903-021-01592-2 PMC8161922

[28] Zhu XZ Zhang JM Wu YF Zhao L. Immunoglobulin G4-related diseases with gingival hyperplasia: a case report. Zhonghua Kou Qiang Yi Xue Za Zhi. 2022;57:867–70. 35970783 10.3760/cma.j.cn112144-20211208-00541

[29] Ike Y Shimizu T Ogawa M. Ossifying fibrous epulis as an IgG4-related disease of the oral cavity: a case report and literature review. BMC Oral Health. 2022;22:4. 35012519 10.1186/s12903-022-02041-4 PMC8744345

[30] Grasso C Giacchero F Crivellari S Bertolotti M Maconi A. A Review on The Role of Environmental Exposures in IgG4-Related Diseases. Curr Environ Health Rep. 2023;10:303–11. 37314670 10.1007/s40572-023-00401-y

[31] Takanashi S Akiyama M Kondo Y Kaneko Y. Avoidance of allergen exposure may ameliorate IgG4-related disease: a case that improved spontaneously after moving house. Int J Rheum Dis. 2024;27:e14892. 37635356 10.1111/1756-185X.14892

[32] Takanashi S Kikuchi J Sasaki T. Lymphadenopathy in IgG4-related disease: a phenotype of severe activity and poor prognosis, with eotaxin-3 as a new biomarker. Rheumatology (Oxford). 2021;60:967–75. 33167029 10.1093/rheumatology/keaa648

[33] Della Torre E Mattoo H Mahajan VS Carruthers M Pillai S Stone JH. Prevalence of atopy, eosinophilia, and IgE elevation in IgG4-related disease. Allergy. 2014;69:269–72. 24266692 10.1111/all.12320 PMC4638385

[34] Zhou J Peng Y Peng L. Serum IgE in the clinical features and disease outcomes of IgG4-related disease: a large retrospective cohort study. Arthritis Res Ther. 2020;22:255. 33097076 10.1186/s13075-020-02338-1 PMC7583198

[35] Khosroshahi A Wallace ZS Crowe JL. International consensus guidance statement on the management and treatment of IgG4-Related Disease. Arthritis Rheumatol. 2015;67:1688–99. 25809420 10.1002/art.39132

[36] Lanzillotta M Della-Torre E Wallace ZS. Efficacy and safety of rituximab for IgG4-related pancreato-biliary disease: a systematic review and meta-analysis. Pancreatology. 2021;21:1395–401. 34244040 10.1016/j.pan.2021.06.009

[37] Khan AA Morrison A Hanley DA. Diagnosis and management of osteonecrosis of the jaw: a systematic review and international consensus. J Bone Miner Res. 2015;30:3–23. 25414052 10.1002/jbmr.2405

[38] Ruggiero SL Dodson TB Aghaloo T Carlson ER Ward BB Kademani D. American Association of oral and maxillofacial surgeons’ position paper on medication-related osteonecrosis of the jaws-2022 Update. J Oral Maxillofac Surg. 2022;80:920–43. 35300956 10.1016/j.joms.2022.02.008

[39] Yim CD An HJ Ahn SK Hur DG Lee HJ. IgG4-related disease presenting as otogenic skull base osteomyelitis. Auris Nasus Larynx. 2021;48:166–70. 32111411 10.1016/j.anl.2020.02.006

