# Supplementary material for: Generalized gingival enlargement, periodontitis, and osteonecrosis of the jaw as manifestations of IgG4-related disease: A rare case report
Source: Medicine (Baltimore). 2025 Jul 11;104(28):e43260. doi: 10.1097/MD.0000000000043260 (PMC12263011; doi:10.1097/MD.0000000000043260)

**Supplementary figure 1.** Intraoral photographs of the patient returned again 9 months after the surgical interventions: no recurrence of gingival hyperplasia was shown.


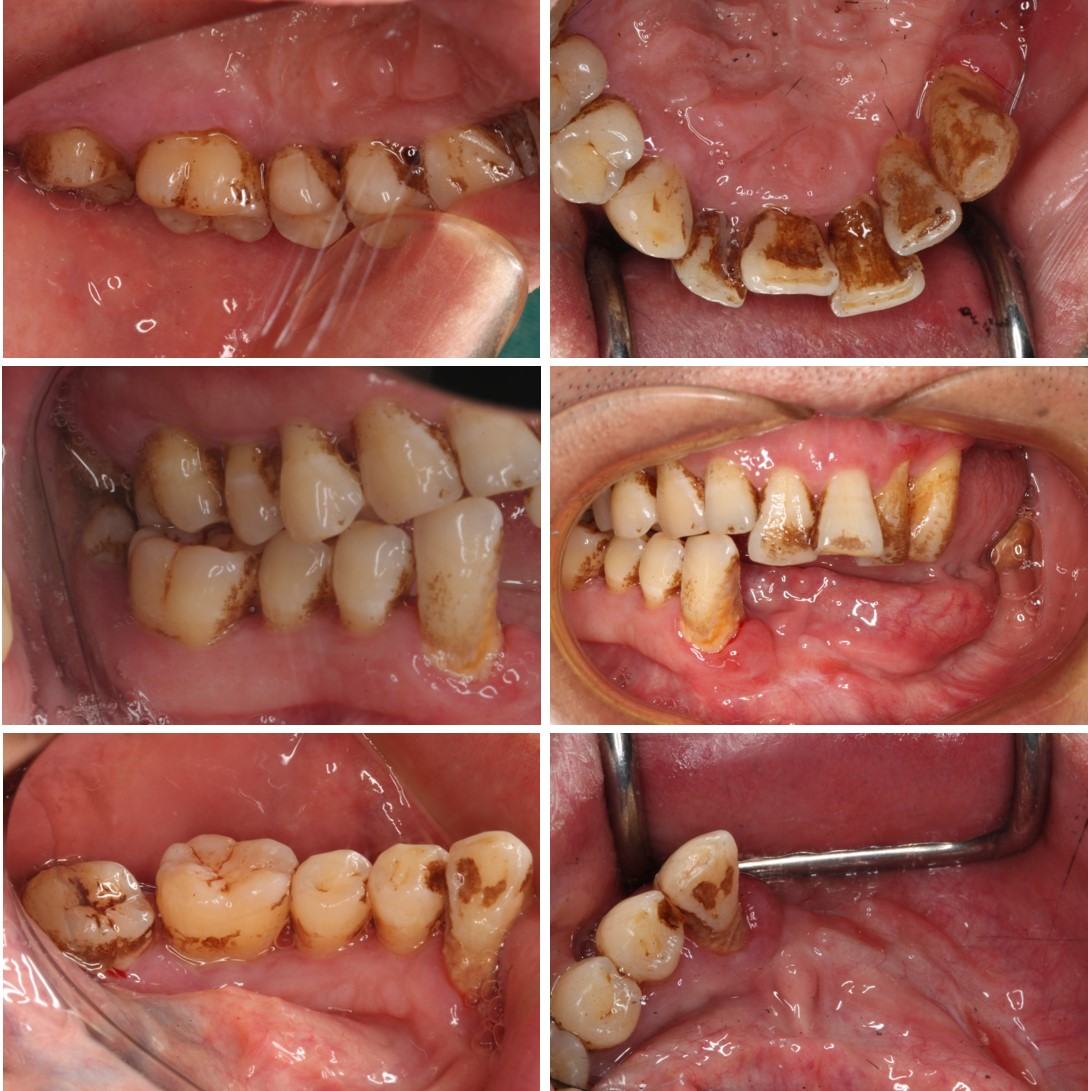

Supplement: Supplementary file 1 [file medi-104-e43260-s001.docx]
